# An Ab Initio Investigation on Relevant Oligomerization Reactions of Toluene Diisocyanate (TDI)

**DOI:** 10.3390/polym14194183

**Published:** 2022-10-05

**Authors:** Ravikumar Thangaraj, Béla Fiser, Xuanbing Qiu, Chuanliang Li, Béla Viskolcz, Milán Szőri

**Affiliations:** 1 Institute of Chemistry, University of Miskolc, Miskolc-Egyetemváros A/2, H-3515 Miskolc, Hungary; 2 Higher Education and Industrial Cooperation Centre, University of Miskolc, H-3515 Miskolc, Hungary; 3 Ferenc Rakoczi II Transcarpathian Hungarian College of Higher Education, 90200 Berehove, Ukraine; 4 School of Applied Science, Taiyuan University of Science and Technology, Taiyuan 030024, China

**Keywords:** dimerization, trimerization, oligomerization, toluene diisocyanate, G3MP2B3

## Abstract

2,4- and 2,6-isomers of toluene diisocyanates (2,4-TDI and 2,6-TDI) are important raw materials in the polyurethane industry. These reactive compounds associate even under ambient conditions to form oligomers, changing the physicochemical properties of the raw material. Kinetically and thermodynamically relevant dimerization reactions were selected based on G3MP2B3 calculations from all possible dimers of phenyl isocyanate using these isocyanates as proxies. As it turned out, only the formation of the diazetidine-2,4-dione ring (11-dimer, uretdione) resulted in a species having an exothermic enthalpy of formation (−30.4 kJ/mol at 298.15 K). The oxazetidin-2-one ring product (1-2-dimer) had a slightly endothermic standard enthalpy of formation (37.2 kJ/mol at 298.15 K). The mechanism of the relevant cyclodimerization reactions was investigated further for 2,4-TDI and 2,6-TDI species using G3MP2B3 and SMD solvent model for diazetidine as well as oxazetidin-2-one ring formation. The formation of the uretdione ring structures, from the 2,4-TDI dimer with both NCO groups in the meta position for each phenyl ring and one methyl group in the para and one in the meta position, had the lowest-lying transition state (Δ^#^*E*_0_ = 94.4 kJ/mol) in the gas phase. The one- and two-step mechanisms of the TDI cyclotrimerization were also studied based on the quasi-G3MP2B3 (qG3MP2B3) computational protocol. The one-step mechanism had an activation barrier as high as 149.0 kJ/mol, while the relative energies in the two-step mechanism were significantly lower for both transition states in the gas phase (94.7 and 60.5 kJ/mol) and in ODCB (87.0 and 54.0 kJ/mol).

## 1. Introduction

Polyurethane (PU) production is increasing rapidly due to its versatile applications in construction blocks, insulating foams, flame retardants in the construction industry, and coatings in the automotive and space industries, and certain compositions are suitable for the use as biomaterials [1,2]. The global PU market was estimated to be 54 billion USD in 2021 and it is forecasted to reach 75.3 billion USD in 2026 [3]. Since mainstream industrial polyurethane production is based on the reaction of isocyanate and polyol, an increasing amount of isocyanate and polyol production is necessary to meet this growing demand. Among the isocyanates, methylene diphenyl diisocyanate (MDI) and toluene diisocyanate (TDI) are produced in large quantities, together comprising about 90% of the total diisocyanate market [4]. TDI is mainly used in the production of flexible polyurethane foams, adhesives, coatings, sealants, and elastomers [5]. Its production starts with the dinitration of toluene, which is a two-stage process. Due to the nitration, a mixture of 2- and 4-nitrotoluene isomers is formed first and this mixture is then further nitrated in a continuous process which mainly results in the formation of 2,4- and 2,6-dinitro-toluene isomers. Subsequent hydrogenation transforms the nitro groups into amine groups forming toluene diamines (TDA) which are treated further and converted to TDI isomers via phosgenation [6]. However, it should be noted that 2,4-TDI and 2,6-TDI are two commercial isomers among the six possible TDI structures and 80/20 mixtures of these isomers have the highest industrial importance [5].

Aromatic isocyanates and diisocyanates are more prone to dimerization, especially in the presence of base catalysts such as trialkylphosphines, aryldialkylphosphines, tris-dialkyl carbamoyl phosphites, hexaalkylphosphortriamides, pyridines and N-substituted guanidines [7,8]. Phosphines, and particularly trialkylphosphine, are highly efficient compared to pyridine in catalyzing the dimerization reaction [9]. No dimer of 2,6-TDI is formed under normal storage conditions, but the isomer 2,4-TDI easily forms a dimer due to the difference in reactivity of the two isocyanate groups [4]. The rate of dimerization of 2,4-TDI is approximately 0.005% per day at 40 °C [4]. Isocyanate dimerization is a reversible reaction, even at moderate temperatures (175 °C); the reversibility of dimers occurs completely and hence it is used as an in-situ isocyanate source for crosslinking hydroxylated polymers in coatings. Querat et al. [10] used the 2,4-TDI dimers as blocked isocyanates and studying their reaction and thermal behavior with polycaprolactone diol.

TDI can also undergo trimerization, which can produce a six-membered ring structure because of its higher reactivity compared to its aliphatic counterparts [8]. Base catalysts such as alkali metal carbonates, alkoxides, oxides and acetates; lead salts; metal naphthenates; tertiary amines; and hydroxides of quaternary ammonium or phosphonium promote this reaction [8,11]. Trimers have the advantage of low volatility, low toxicity, high functionality, corrosion resistance, and high thermal stability, therefore they are used to prepare many branched or crosslinked polymers [8]. Generally, both dimerization and trimerization occur together, but the latter is preferred at higher temperatures [8].

TDI dimers and trimers possess higher NCO functionality in their moieties. They inhibit the volatility of TDI monomers [12], which avoids the high toxicity of TDI vapor [13] and imparts better thermal and mechanical properties. The dimer is suitable for the preparation of linear polyurethane materials, whereas the trimer is used in the crosslinking of PU materials, e.g., coating applications [14].

Moreover, the six-membered trimer ring in the prepolymer remains stable up to 200 °C due to an irreversible trimerization process [13]. The prepolymer is a mixture composed of several oligomers, such as trimers (containing three NCO groups), pentamers (containing four NCO groups), heptamers (containing five NCO groups), or higher-molecular-weight oligomers. Among these, the trimer is the most desired while the presence of higher-molecular-weight oligomers is not favorable in the polyisocyanate prepolymer. The high content of the higher-molecular-weight oligomers may increase the viscosity of the polymer or even gelate and weaken its curing properties [13]. Experimental studies have been reported on the oligomerization of TDI. For example, Guo et al. [13] synthesized TDI-based polyisocyanate prepolymer, under the optimal conditions of 0.5 wt% Mannich base catalyst, at 40–50 °C, and a reaction time of 60 min, and verified the step-growth polymerization by mathematic simulation. Furthermore, the TDI dimer has been successfully prepared in water in the presence of a surface-active agent and a catalyst of trimethylamine or tributyl phosphine [15]. This led to the formation of the aqueous dispersion of the dimer and was able to provide a stable composition, but with heating, the dimer regenerated the diisocyanate monomer to form a useful high polymer [15]. Wang et al. [14] synthesized the TDI/hexamethylene diisocyanate (HDI) hybrid trimer by the cyclo-oligomerization of TDI and HDI using a 3-(trimethylammonio)propyl carbonate catalyst, resulting in a high degree of polymerization with excellent solvent tolerance for coating applications.

Davis [16] studied the rates of dimerization and trimerization of 2,4-TDI at temperatures ranging from 40 °C to 100 °C and reported activation energies of 87.9 kJ/mol and 66.9 kJ/mol for the initial dimerization and trimerization process, respectively. It was reported that the rate of trimerization remained essentially constant over the temperature range and the rate of dimerization decreased gradually to zero.

Recently, ab initio methods were also employed to investigate the reactivity and reaction pathways of aliphatic isocyanates, as reported by Okumoto and Yamabe [17]. They studied the cyclotrimerization of methyl isocyanate in the presence of a base catalyst using density functional theory (DFT). Gibb and Goodman [18] studied the dimerization and trimerization of methyl isocyanate and phenyl isocyanate in the gas phase and toluene phase using B3LYP/6-31G** and M05-2X/6-31G** level of theories. They found that the activation barrier was relatively lower in the case of M05-2X/6-31G** for both the dimer (105 kJ/mol) and the trimer (167 kJ/mol for one-step formation and 136.2 kJ/mol for two-step formation). Recently, Yunfei et al. [19] studied the role of acetate anions in the trimerization reaction of aromatic isocyanates using experimental and computational methods. They used the TPSS-D3/def2-TZVP level of theory to study this reaction in solvents such as toluene and tetrahydrofuran (THF). Furthermore, Uchimaru et al. [20] performed a computational study on the thermochemical properties of isocyanurate molecules with various alkyl substituents using the ONIOM(CCSD(T)/cc-pVTZ:B3LYP-GD3/cc-pVTZ) and ONIOM(CCSD(T)/cc-pVTZ:MP2/cc-pVTZ) quantum chemical approaches. Their results suggest that the degree of deformation of the isocyanurate ring is related to the substituents on the nitrogen atoms and the inter-substituent attractive dispersion interactions, which also control the thermal stability.

Apart from the gas-phase reaction, reactions in solvents play a huge role in industries in terms of altering the activation barrier. In the case of TDI, it is soluble in organic solvents such as diethyl ether, carbon tetrachloride, benzene, monochlorobenzene, dichlorobenzene, and kerosene [4]. Among these solvents, ortho-dichlorobenzene (ODCB) is primarily used in the production of MDI and TDI [4].

This work aims to further explore the reaction mechanisms of dimerization of 2,4-TDI and 2,6-TDI using an accurate and robust G3MP2B3 model chemistry (which has an average absolute deviation of 5.23 kJ/mol for 299 energies of the G2/97 test set [21]) and implicit SMD solvent model, a combination which has already been successfully used for similar systems [6,22,23]. Furthermore, it also studies the mechanism for the formation of the TDI trimer based on G3MP2B3 and B3LYP/6-31G(d) calculations, which are described in detail in the next section.

## 2. Computational Methods

The G3MP2B3 composite method [21] was employed to study the dimerization reaction of TDI; this method is implemented in the Gaussian09 program package [24]. The method starts with geometry optimization using the B3LYP [25,26]/6-31G(d) [27,28,29] level of theory with a “tight” convergence criterion (thresholds: maximum force = 0.000015 a.u., RMS force = 0.000010 a.u., maximum displacement = 0.000060 a.u. and RMS displacement = 0.000040 a.u.) [30]. Frequency calculations were also carried out using the aforementioned level of theory to get the zero-point corrected relative B3LYP/6-31G(d) energy (Δ*E*_0,B3LYP/6-31G(d)_) and the harmonic wavenumbers were scaled by a factor of 0.96 [21]. Further calculations were required to refine the electronic energy generated and the corrections were made with single-point calculations carried out using QCISD(T)/6-31G(d) (including MP2/6-31G(d) level of theory) and MP2/GTMP2 levels of theory based on B3LYP/6-31G(d) geometries.

Normal mode analysis was performed on the optimized structures at the B3LYP/6-31G(d) level of theory to confirm that the molecules and molecular complexes corresponded to the minimum potential energy surface (PES), while all transition states were first-order saddle points of the PES. Furthermore, the molecular motion along the imaginary wavenumbers of the transition state (TS) structures was visually inspected by means of the GaussView 6 [31] program. For further justification, intrinsic reaction coordinate (IRC) [32] calculations were carried out to map the minimum energy pathways. Finally, the thermochemical properties such as zero-point corrected relative energy (Δ*E*_0,G3MP2B3_), relative enthalpy (Δ*H*^0^_G3MP2B3_), standard enthalpy of formation (Δ_f,298.15K_*H*^0^(g)), and relative Gibbs free energy (Δ*G*^0^_G3MP2B3_) were calculated using the G3MP2B3 composite method. The Δ_f,298.15K_*H*^0^(g) readings were obtained with an atomization scheme (AS) using the standard enthalpy of formation for atoms given at CCCBDB [33]. The accuracy of the protocol for measuring the enthalpy of formation has been proven for similar systems [6,23].

The polarizable continuum model (PCM) with the radii and non-electrostatic terms of Truhlar et al. (SMD) [34] were used to implicitly mimic the surrounding ortho-dichlorobenzene (ODCB) environment of the dimerization reaction. In the SMD model, all gas-phase structures are re-optimized with the SMD treatment of the ODCB, and it is fully integrated into the G3MP2B3 protocol. The G3MP2B3 composite thermochemistry method is not usable for assessing trimer structures because of their molecular size. Therefore, two sets of calculations were assigned for these systems, namely, the entire system was treated at the B3LYP/6-31G(d) level of theory to include the effect of the methyl and isocyanate groups situated away from the reaction center (which did not undergo covalent bond rearrangements in the reaction), and the B3LYP/6-31G(d) level of theory and the consequent G3MP2B3 calculations were carried out on the truncated system prepared in such a way that all methyl and isocyanate groups situated away from the reaction center were replaced by a hydrogen atom (truncated system). The comparison of the whole and the truncated systems was used as the measure of the side chain and the corresponding solvent effects, while the method dependency of the results was corrected by the relative energy difference between the B3LYP/6-31G(d) and G3MP2B3 results. With this computational protocol, quasi-G3MP2B3 quality results are expected, notated as “qG3MP2B3” in this article. A comparison of qG3MP2B3 and G3MP2B3 is shown in the results and discussion section. Trimerization electronic energy was calculated using the following equation:

$$E_{\mathrm{qG3MP2B3}}=\left(E_{\mathrm{whole},B3LYP}-E_{\mathrm{truncated},B3LYP}\right)+E_{\mathrm{truncated},G3MP2B3}$$

## 3. Results and Discussion

### 3.1. Thermochemistry of the Phenyl Isocyanate Covalent Dimers

**Figure 1:**
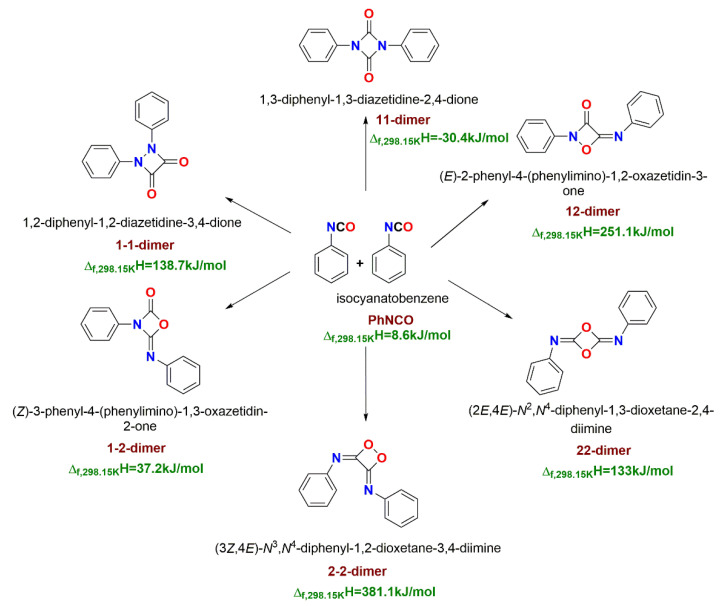
Reaction scheme for the cyclodimerization of phenyl isocyanate. The gas-phase G3MP2B3 standard enthalpy of formation values are also provided (green values).

To model all relevant TDI homocyclization reactions, phenyl isocyanate (PhNCO) can be used as a proxy for determining all the covalent dimer structures of our interest. As shown in Figure 1, phenyl isocyanate undergoes covalent dimerization through six different reaction pathways and forms six different products. Standard enthalpy of formation has been calculated for all six products at the thermodynamic standard condition of 298.15 K and 1 atm pressure. Among these structures, four-membered ring structures of exothermic diazetidine-2,4-dione (11-dimer, uretdione) and endothermic oxazetidin-2-dione (1-2-dimer) have the lowest standard enthalpy of formation of −30.4 kJ/mol and 37.2 kJ/mol (both obtained at G3MP2B3), respectively. Other covalent dimers have a significantly higher enthalpy of formation (>130 kJ/mol) which cannot be accessed under industrially relevant conditions. Hence these two structures were considered for the further study of TDI homocyclization.

### 3.2. TDI Cyclodimerization

To elucidate the effect coming from the additional isocyanate and methyl group of TDI on the uretdione (11-dimer) and oxazetidin-2-one (1-2-dimer) dimer stabilization, all the possible six combinations of 2,4- and 2,6-TDI dimer formation were investigated. For the book-keeping, the TDI dimers were denoted as 11-TDI-dimer and 1-2-TDI-dimer, and their structures were denoted as “x_monomer1_monomer2_pos”, where x represents 11-TDI-dimer or 1-2-TDI-dimer, monomer1 and monomer2 are either 2,4-TDI or 2,6-TDI, and pos indicates the interacting position of the isocyanate group.

**Figure 2:**
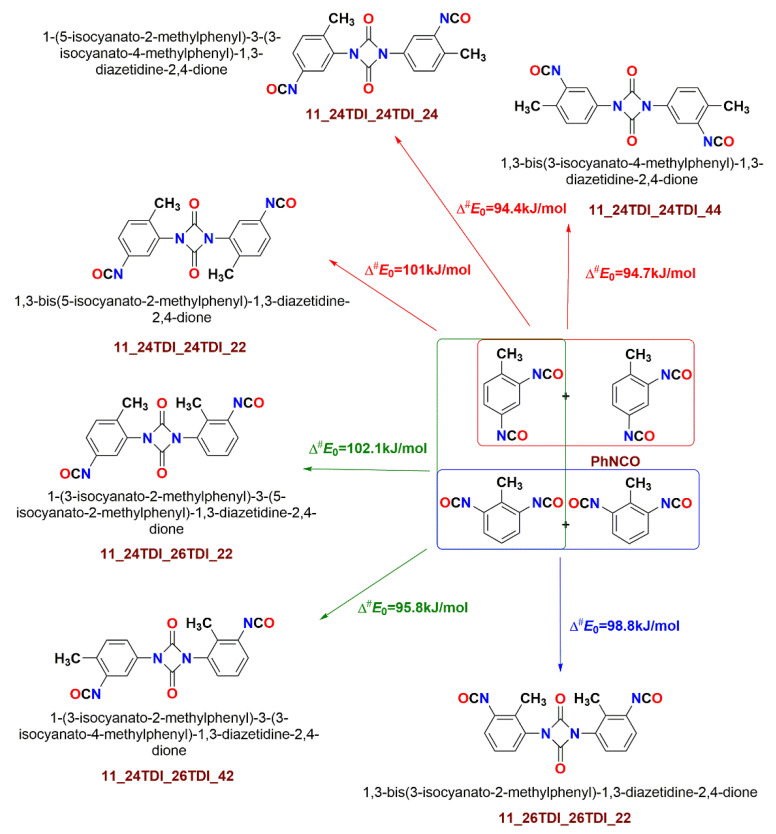
Reaction scheme for the dimerization of TDI isomers via homocyclization leading to diazetidines. The gas-phase activation energies (Δ^#^*E*_0_) were computed according to the G3MP2B3 protocol.

Nitrogen in the first monomer forms a bond with carbon in the second monomer, and nitrogen in the second monomer forms a bond with carbon in the first monomer, which is denoted as 11-TDI-dimer (uretdione). The bond formation between nitrogen in the first monomer and carbon in the second monomer and carbon in the first monomer and oxygen in the second monomer is denoted as 1-2-TDI-dimer. The corresponding 11-TDI-dimer structures are shown in Figure 2. TDI homocyclization was studied under four different conditions (pressure was kept constant at 1 atm), specifically at the standard temperature of 298.15 K and the industrially more relevant elevated temperature of 423.15 K in both the gas phase and ODCB. The results obtained are tabulated in Table 1.

Comparing the reaction enthalpy for the type ‘11’ PhNCO dimer (uretdione) formation (Δ*H*^0^_G3MP2B3_ = −47.6 kJ/mol at 298 K) with the corresponding TDI dimer formations, there was a slight alteration around PhNCO value ranging between −35.7 kJ/mol and −50.2 kJ/mol, as shown in Table 2. A similar trend was also observed for the reaction enthalpy of the 1-2-dimers (20.0 kJ/mol vs. 16.0–27.4 kJ/mol). As has been seen in the case of phosgenation of TDA [6], the solvent effect on the reaction enthalpies can be described as a linear relationship: Δ*H*^0^_G3MP2B3_(ODCB) = 1.11Δ*H*^0^_G3MP2B3_(gas)—6.5 kJ/mol, although the fitted parameters are different here (system dependent). According to this, the presence of the solvent makes the reactions more exothermic than they are in the gas phase. The activation enthalpies also decreased due to the slightly polar ODCB environment; the activation enthalpies varied from 93.3 kJ/mol to 124.7 kJ/mol for the gas phase and 85.4 kJ/mol to 103.3 kJ/mol for ODCB. The 1-2 transition states tended to be as high as 100 kJ/mol, regardless of which arrangements and which TDI isomers were involved in the dimerization. Therefore, the formation of the uretdione ring (11 dimer) is kinetically more favorable than the oxazetidin-2-one ring (1-2 isomer), and the reaction resulting in a uretdione ring is discussed further below. Boros et al. reported that the -NCO group in the para-position is more reactive for methylene diphenyl diisocyanate (MDI) [35] and a similar trend can be observed in TDI as well. 

Among the formation of the diazetidine ring structures, the neat 2,4-TDI dimer with both NCO groups in the meta position for each phenyl ring and one methyl group in para and one in the meta position has the lowest-lying transition state (Δ^#^*E*_0_ = 94.4 kJ/mol) in the gas phase (noted as 11_24TDI_24TDI_24 in Figure 2). This activation energy is just slightly lower than with the highly symmetrical 2,4-TDI dimer (Δ^#^*E*_0_ = 94.7 kJ/mol), which has both NCO groups in the meta position and both methyl groups in the para position (noted as 11_24TDI_24TDI_44). The Δ^#^*E*_0_ for the neat 2,6-TDI dimer with ortho NCO and meta CH_3_ groups are 4.1 kJ/mol higher (98.8 kJ/mol) than that of the previously mentioned TS. 

This order can be different in ODCB or at different temperatures, as well as if other thermodynamical properties are considered; therefore, only these transition state structures will be discussed later in detail, while the activation energy for the remaining dimers is about 100 kJ/mol, therefore they may play only minor roles in the TDI dimerization.

**Figure 3:**
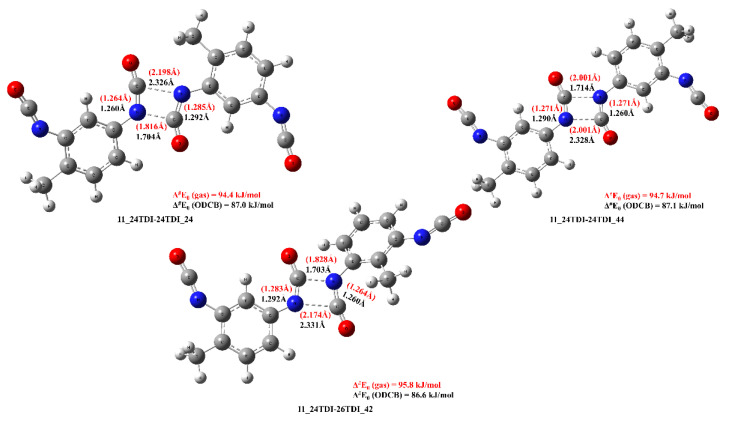
The low-lying transition state structures of TDI dimerization in the gas phase (in red) and in ODCB (in black) obtained at the B3LYP/6-31G(d) level of theory.

As seen in Figure 3, these low-lying four-centered transition state structures share high similarities with each other, namely distances of the forming C-N bonds are about 1.70 Å and 2.33 Å in ODCB, while this asymmetry in C-N distances is less pronounced in the gas phase (1.80 Å and 2.2 Å) and the TS of the 11_24TDI-24TDI_44 is C_2h_ symmetric with the C-N distances of 2.001 Å.

Assuming chemical equilibrium between reactants and dimer product, the Gibbs free energy values (last two columns in Table 1) can be turned into equilibrium constants (K). Since only structures in ODCB have industrial purposes, their equilibrium constants are presented in Table 2 at two different temperatures. If the process is purely thermodynamic-driven, at room temperature the formation of the type ‘44’ dimer is thermodynamically preferred, and other dimer structures are only presented as minor in the mixture. At elevated temperatures, the entropy contribution to the Gibbs free energy becomes dominant and therefore the equilibrium will be more on the monomer’s side.

### 3.3. Validation of the qG3MP2B3 Protocol

**Figure 4:**
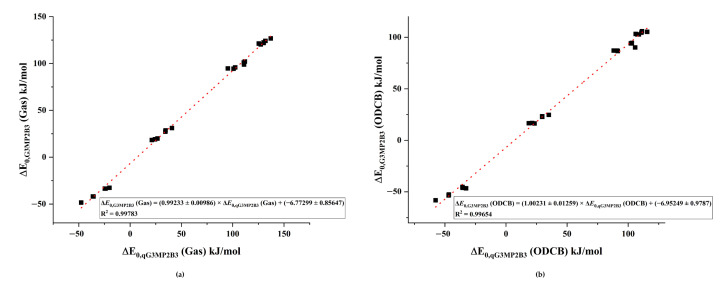
Comparison between the relative energies of the dimers computed using qG3MP2B3 and G3MP2B3 in the (**a**) gas phase and (**b**) ODCB. Fitted plots are marked by dotted lines.

Since the G3MP2B3 procedure is not feasible for cyclotrimerization, a modified G3MP2B3 protocol is required which still conserves the robustness of G3MP2B3, therefore qG3MP2B3 was introduced in the computational methods section. A comparison of the qG3MP2B3 and G3MP2B3 relative energies for all cyclodimerization reactions presented in Figure 2 is shown in Figure 4, for both in (a) the gas phase and (b) ODCB. As can be seen from the figures, the qG3MP2B3 energies were in a nearly ideal linear relationship with the G3MP2B3 in both phases (both slopes are close to unity). The R^2^ values were 0.998 and 0.997 and the magnitudes of the intercepts were −6.8 kJ/mol and −7.0 kJ/mol in the gas phase and ODCB, respectively. This suggests that qG3MP2B3 could be a reasonable alternative to G3MP2B3 for cyclotrimerization studies.

### 3.4. Cyclotrimerization Reactions of 2,4-TDI

**Figure 5:**
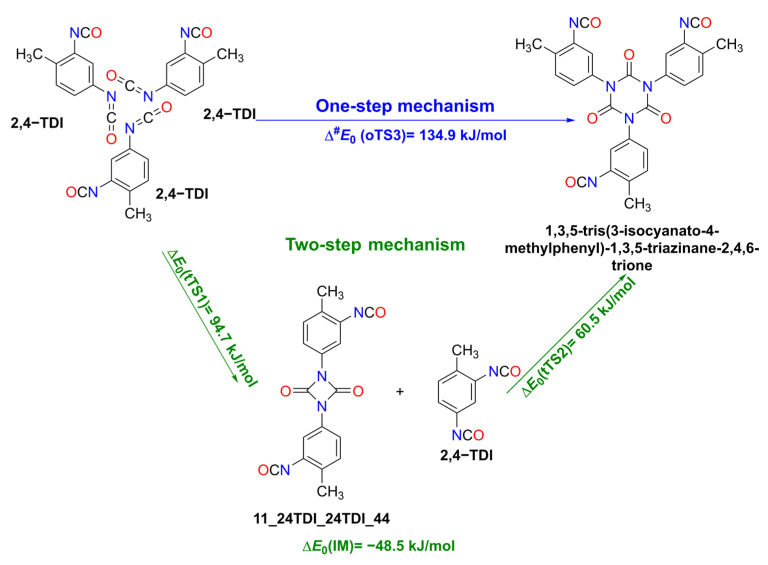
The one-step and two-step mechanisms resulting in a 2,4-TDI trimer. The qG3MP2B3 zero-point corrected relative energies are also presented.

Oligomerization, which leads to the formation of cyclic TDI trimers, can occur via two different mechanisms: a one-step and a two-step process. As Figure 5 shows, the one-step addition reaction of three non-covalently interacting 2,4-TDI monomers result in the formation of a six-membered ring (trimer). On the other hand, in the two-step mechanism, addition reactions of the TDI monomers take place gradually in such a way that the TDI dimer forms first, as mentioned in the previous section, and the intermediate stage, which consists of this dimer and a monomer (IM), then transforms into the final trimer product (Figure 5, green). Also, as a consequence of the previous section, only 2,4-TDI trimers were studied since substitution plays only a minor role, as we will also see later when the truncated and whole systems are compared energetically.

**Figure 6:**
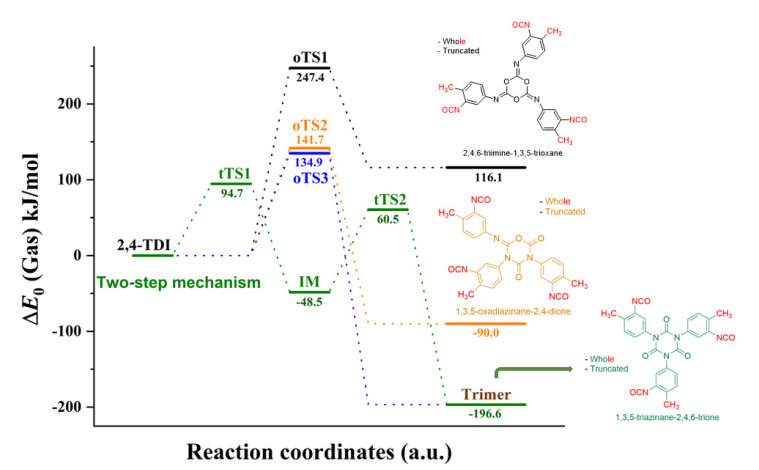
Zero-point energy profile for the trimerization of TDI in the gas phase. The energy values were obtained using the qG3MP2B3 protocol.

**Figure 7:**
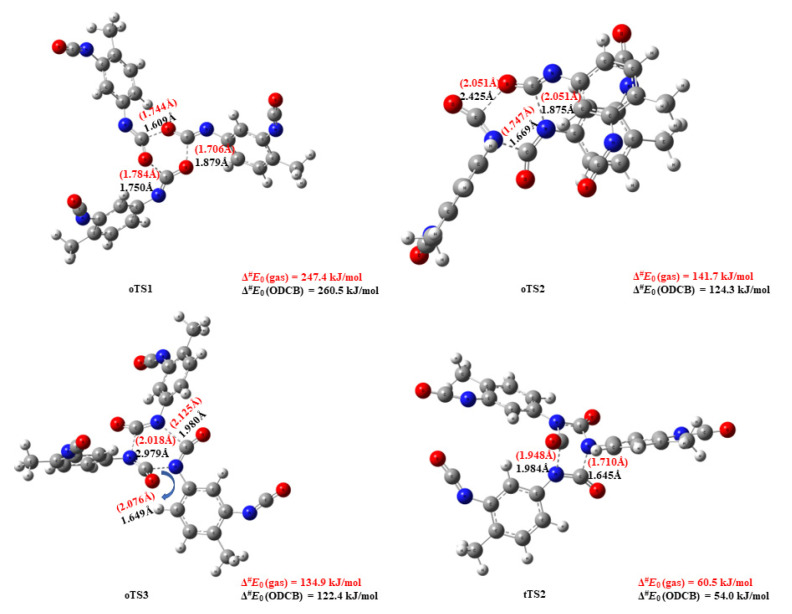
Transition state structures of the trimerization of TDI in the gas phase and the ODCB environment. The energy values were obtained using the qG3MP2B3 protocol.

2,4-TDI can form three ring types of trimers based on the relative orientation of NCO ring-building blocks, as shown in Figure 6. When a carbon from an NCO group covalent bonds to the other NCO’s oxygen to form the ring, it results in the formation of a triamine-substituted 1,3,5-trioxane structure in the one-step mechanism. As is clear from the potential energy surface, this trioxane undergoes an extremely endothermic formation (Δ_r_*E*_0_ = 116.1 kJ/mol) with high activation energy (Δ^#^*E*_0_(oTS1) = 247.4 kJ/mol) making it unlikely to occur. The corresponding transition state structure (oTS1) with short, similar critical distances (ca. 1.7 Å) is presented in Figure 7. The bonding of nitrogen and carbon of different NCO groups gives another possibility for ring structures. If two NC bonds and a CO bond form between the TDI monomers, the product is oxadiazinane-2,4-dione (imino-oxadiazinedione) shown in Figure 6 (in orange). This reaction has an activation energy that is roughly 100 kJ/mol lower (Δ^#^*E*_0_(oTS2) = 141.7 kJ/mol) and it is also exothermic (Δ_r_*E*_0_ = −90.0 kJ/mol). The differences in critical parameters of the TS structure are significantly larger (Figure 7): the distance is 1.747 Å for one of the forming NC, and 2.051 Å for the other NC and the CO as well. Finally, if only simultaneous formation of three NC bonds occurs, then the relative energy of the TS is 134.9 kJ/mol (oTS3), giving an extremely exothermic product, substituted 1,3,5-triazinane-2,4,6-trione, better-known isocyanurate (Δ_r_*E*_0_ = −196.6 kJ/mol), the exothermicity of which is in line with the calculated triphenyl isocyanurate formation using ONIOM model. [20]

Figure 6 contains the whole and the truncated system. As the name suggests, the whole represents the entire system, highlighted in red and other colors (black, orange, and green), and truncated denotes the system without the excess -NCO group and methyl group (only highlighted in black, orange, and green).

Given that this reaction has the lowest activation energy and highest exothermicity, we also investigated its two-step mechanism counterpart, and the corresponding energy profile is also shown in Figure 6. In this mechanism, the first transition state structure (tTS1) is identical to the TS in the dimer section, notated as 11_24TDI_24TDI_44, therefore its structure is not discussed here in detail. However, the transition state (tTS2) from the intermediate structure (IM) towards the trimer structure is depicted as tTS2 in Figure 7. The tTS2 is a four-centered transition state in which the C-N bond is being broken, while two new C-N bonds are being formed with relatively small C-N distances (1.7 and 1.9 Å), resulting in a six-membered isocyanurate trimer. The relative energy of the IM structure is mildly negative (−48.5 kJ/mol) and the energy of the second transition state (60.5 kJ/mol) is lower by 34.2 kJ/mol than that of the first one. These energies are all obtained by using the qG3MP2B3 protocol, since G3MP2B3 was not feasible for the whole trimer calculation due to the computational demand of the QCISD(T)/6-31G(d) single point. Therefore, a truncated system was calculated using G3MP2B3 and the correction to the whole system was carried out at the B3LYP/6-31G(d) level of theory in such a way that both whole and truncated systems were computed at this level of theory (the definition of the systems are color coded in Figure 6). This also provides an opportunity to compare the relative DFT energies of the two systems. As it turned out, the relative energy contribution of the substitution was −4.1 ± 0.7 kJ/mol in the gas phase and it was lower in ODCB (−2.6 ± 0.5 kJ/mol) with the ideal slope of 1, therefore the truncation approximation seems to be valid. Again, the effect of the presence of the ODCB environment can be represented by a linear relationship, which means a small shift towards lower relative energies; they are not discussed here, but the calculated thermochemical properties of all the above-mentioned structures are collected in Table 3.

## 4. Conclusions

In this work, cyclo-oligomerization of 2,4- and 2,6-TDI was computationally investigated. Based on our extended thermochemical evaluation, two types of dimer products were selected as plausible to form: diazetidine-2,4-dione (11-dimer) and oxazetidin-2-one (1-2-dimer). To get to each product, six transition states are possible. Among these twelve transition states, 11_24TDI_24TDI_24, 11_24TDI_24TDI_44, and 11_24TDI_26TDI_42 structures have the lowest-lying activation barrier, due to the highly reactive NCO group in the para position, which makes them favorable.

For cyclotrimerization, two types of reaction mechanism were proposed: a one-step and two-step process. Three one-step mechanisms were found, which can be distinguished by the relative orientation of the NCO group of the monomers, their energy profiles was explored. Among the three transition states, oTS3 had the lowest activation barrier, resulting in the exothermic formation of the substituted isocyanurate (1,3,5-triazinane-2,4,6-trione) ring. The same product can form via a two-step mechanism, within which the structurally highly stabilized uretdione dimer (11_24TDI_24TDI_44) and the TDI monomer react. The two-step process had the lowest activation barrier compared to the three activation energies achieved from the one-step mechanism. The qG3MP2B3 computational protocol was utilized for the trimer study and it revealed a strong correlation with the truncated system, meaning that the methyl and NCO groups far from the reaction center did not change the energy profile significantly, even if they were directly connected to them via the aromatic ring.

## Figures and Tables

**Table 1:** G3MP2B3 thermochemical properties calculated in the gas phase and in ortho-dichlorobenzene (ODCB), including zero-point corrected relative energies (Δ*E*_0,G3MP2B3_), relative enthalpies (Δ*H*^0^_G3MP2B3_), and relative Gibbs free energies (Δ*G*^0^_G3MP2B3_, *p* = 1 atm).

		Δ*E*_0,G3MP2B3_		Δ*H*^0^_G3MP2B3_				Δ*G*^0^_G3MP2B3_			
Solvent		-	ODCB	-	ODCB	-	ODCB	-	ODCB	-	ODCB
T in K		0		298.15		423.15		298.15		423.15	
11_24TDI_24TDI_22	TS	101.0	94.0	99.8	92.4	100.4	93.0	155.4	149.9	178.7	173.6
11_24TDI_24TDI_24	TS	94.4	87.0	93.3	85.9	93.8	86.4	147.7	140.3	170.5	162.5
11_24TDI_24TDI_44	TS	94.7	87.1	94.2	86.1	94.8	86.6	144.5	141.3	165.5	164.4
11_24TDI_26TDI_22	TS	102.0	94.5	100.7	93.0	101.3	93.5	156.9	150.6	180.4	174.6
11_24TDI_26TDI_42	TS	95.8	86.6	94.7	85.4	95.3	85.9	150.5	141.9	173.8	165.1
11_26TDI_26TDI_22	TS	98.8	90.1	97.0	88.0	97.6	88.3	156.1	147.6	180.8	172.1
1-2_24TDI_24TDI_22	TS	121.9	102.7	120.4	101.1	120.9	101.4	178.1	158.1	202.2	182.3
1-2_24TDI_24TDI_24	TS	120.3	102.8	118.8	100.8	119.3	101.1	176.0	158.7	199.8	182.9
1-2_24TDI_24TDI_44	TS	121.3	103.2	120.0	101.3	120.5	101.6	176.0	160.0	199.5	184.5
1-2_24TDI_26TDI_22	TS	124.1	104.4	122.6	102.9	123.0	103.2	179.7	160.0	203.6	183.8
1-2_24TDI_26TDI_42	TS	122.5	105.8	120.6	103.3	121.1	103.0	179.5	164.6	204.1	187.5
1-2_26TDI_26TDI_22	TS	126.7	105.2	124.7	102.4	125.3	102.7	183.7	165.7	208.3	192.1
11_24TDI_24TDI_22	Product	−33.6	−45.4	−36.3	−47.6	−36.0	−47.3	25.6	12.0	51.5	36.9
11_24TDI_24TDI_24	Product	−42.0	−52.7	−44.4	−55.0	−44.2	−54.7	16.1	5.8	41.5	31.3
11_24TDI_24TDI_44	Product	−48.5	−58.1	−50.2	−59.8	−49.9	−59.5	6.3	-2.9	29.9	20.9
11_24TDI_26TDI_22	Product	−33.6	−46.1	−36.3	−48.4	−36.1	−48.1	26.5	10.7	52.9	35.4
11_24TDI_26TDI_42	Product	−42.0	−53.5	−44.6	−55.7	−44.3	−55.4	17.2	2.9	43.1	27.4
11_26TDI_26TDI_22	Product	−32.7	−46.6	−35.7	−49.3	−35.4	−49.0	28.4	11.5	55.3	37.0
1-2_24TDI_24TDI_22	Product	27.2	22.7	24.2	19.9	24.4	20.2	85.5	80.9	111.2	106.4
1-2_24TDI_24TDI_24	Product	19.0	16.7	16.0	13.8	16.3	14.1	76.7	74.8	102.1	100.3
1-2_24TDI_24TDI_44	Product	18.3	16.5	15.5	13.6	15.7	14.0	75.2	75.1	100.3	100.9
1-2_24TDI_26TDI_22	Product	28.6	23.3	25.6	20.5	25.8	20.8	86.6	81.3	112.2	106.8
1-2_24TDI_26TDI_42	Product	19.9	16.3	16.6	13.0	16.9	13.4	78.6	76.2	104.6	102.7
1-2_26TDI_26TDI_22	Product	31.0	24.6	27.4	21.1	27.7	21.5	90.9	84.4	117.5	110.9

**Table 2:** Reaction Gibbs free energies (Δ_r_*G*^0^) and the negative logarithm of equilibrium constant (pK = −logK) of the type ‘11’ TDI dimerization resulted in different uretdione formation calculated at the G3MP2B3 level of theory.

Species	T = 298.15 K		T = 423.15 K	
	Δ_r_*G*^0^ (kJ/mol)	pK	Δ_r_*G*^0^ (kJ/mol)	pK
11_24TDI_24TDI_22	12.0	2.1	36.9	4.6
11_24TDI_24TDI_24	5.8	1.0	31.3	3.9
11_24TDI_24TDI_44	−2.9	−0.5	20.9	2.6
11_24TDI_26TDI_22	10.7	1.9	35.4	4.4
11_24TDI_26TDI_42	2.9	0.5	27.4	3.4
11_26TDI_26TDI_22	11.5	2.0	37.0	4.6

**Table 3:** Thermochemical properties calculated in the gas phase and ortho-dichlorobenzene (ODCB), including zero-point corrected relative energies (Δ*E*_0,qG3MP2B3_), relative enthalpies (Δ*H*^0^_qG3MP2B3_), and relative Gibbs free energies (Δ*G*^0^_qG3MP2B3_).

	Δ*E*_0,qG3MP2B3_ (kJ/mol)		Δ*H*^0^_qG3MP2B3_ (kJ/mol)				Δ*G*^0^_qG3MP2B3_ (kJ/mol)			
Solvent	-	ODCB	-	ODCB	-	ODCB	-	ODCB	-	ODCB
T (K)	0		298.15		423.15		298.15		423.15	
Reactant	0.0	0.0	0.0	0.0	0.0	0.0	0.0	0.0	0.0	0.0
oTS1	247.4	260.5	246.3	259.7	248.3	261.9	350.4	364.8	393.8	408.4
oTS2	141.7	124.3	141.8	124.0	143.9	126.1	246.3	235.1	289.7	281.3
oTS3	134.9	122.4	136.0	122.4	138.3	124.4	236.2	230.1	277.8	275.0
IM	−48.5	−58.1	−50.2	−59.8	−49.9	−59.5	6.3	−2.9	29.9	20.9
tTS2	60.5	54.0	56.2	49.4	57.0	50.3	178.4	171.9	229.6	223.1
substituted 1,3,5-trioxane	116.1	125.3	112.8	121.6	113.8	122.6	220.8	234.2	265.9	281.2
substituted iminooxadiazinedione	−90.0	−105.4	−94.2	−109.8	−93.4	−109.0	23.2	11.2	72.3	61.8
substituted isocyanurate	−196.6	−227.9	−200.8	−232.0	−200.2	−231.5	−84.3	−114.4	−35.5	−65.1

## Data Availability

The data presented in this study are available on request from the corresponding author. The data are not publicly available due to the policy of the University of Miskolc.
